# Toward an Understanding of Cancer as an Issue of Social Justice: Perspectives and Implications for Oncology Nursing

**DOI:** 10.3390/curroncol32020104

**Published:** 2025-02-12

**Authors:** Tara C. Horrill, Scott M. Beck, Allison Wiens

**Affiliations:** 1 College of Nursing, Rady Faculty of Health Sciences, University of Manitoba, 89 Curry Place, Winnipeg, MB R3T 2N2, Canada; 2 BC Cancer Agency, University of British Columbia, 601 W Broadway, Vancouver, BC V5Z 4C2, Canada; 3 CancerCare Manitoba, 675 McDermot Avenue, Winnipeg, MB R3E 0V9, Canada

**Keywords:** health equity, healthcare disparities, health services accessibility, professional practice, social justice, oncology nursing, nursing advocacy

## Abstract

Within the fields of oncology practice and research, cancer has historically been and continues to be understood as primarily biologically produced and physiologically driven. This understanding is rooted in biomedicine, the dominant model of health and illness in the Western world. Yet, there is increasing evidence of inequities in cancer that are influenced by social and structural inequities. In this article, we propose that cancer-related inequities ought to be seen as issues of social justice, and, given nursing’s longstanding commitments to social justice, they ought to be a priority for oncology nurses. Using a social justice lens, we highlight potential social injustices in the form of inequities in cancer outcomes and access to cancer care across the cancer continuum. Our intention is not to provide an exhaustive review of evidence, but to provide our perspective, adding to the dialogue surrounding health equity and cancer while shifting the narrative away from an understanding of cancer inequities as stemming from “lifestyle” and “behavioural” choices. We conclude by exploring the implications of considering cancer inequities as social injustices for nursing practice.

## 1. Introduction

Within the fields of oncology practice and research, cancer has historically been and continues to be understood as primarily biologically produced and physiologically driven [1]. This understanding is rooted in biomedicine, the dominant model of health and illness in the Western world. Within the biomedical model, individuals are generally understood as the source of health and illness, abstracted from any social, economic, political, or other contextual influences, while reducing the focus of healthcare to the treatment of organs and bodily systems [2,3,4]. Renowned social epidemiologist Nancy Krieger articulates the biomedical model as the taken-for-granted approach to understanding cancer:

“*Prioritizing the micro over the macro, both ideologically and technically, the biomedical model simultaneously (i) focuses on the physical, chemical, and biological causes of disease, and (ii) renders invisible how the societal context simultaneously shapes disease rates and the way their causes are conceptualized and analyzed, and by whom. If any social variables appear, they do so as individual risk factors and behavioral choices, framed by the complementary and equally individualistic lifestyle theory. Health inequities receive scant attention. Instead, observed physiological or other biological differences between social groups are largely recast as a matter of intrinsic (also known as genetic) difference, especially for race and ethnicity*”.

[1] (p. 112; emphasis added)

Scanning the tables of contents of oncology journals, lists of funded cancer research projects, and conference agendas confirms the dominance Krieger speaks of. Yet, despite significant biomedically driven advancements in the early detection and treatment of cancer, not all groups benefit from these advances. Indeed, there is increasing evidence that cancer outcomes are influenced by social and structural inequities, and evidence that advances in the field of oncology have served to worsen cancer-related inequities. In this discussion paper, we aim to add to the dialogue surrounding health equity and cancer by proposing that cancer-related inequities ought to be seen as issues of social justice, and, given nursing’s longstanding commitments to social justice [5,6], they ought to be a priority for oncology nurses. We begin by briefly exploring the concept of social justice and its relevance to nursing, and then provide examples of cancer-related inequities to illustrate social injustices. We conclude by discussing the implications of understanding cancer as an issue of social justice within oncology nursing practice.

## 2. A Social Justice Primer

Social justice has been described as the “*morally proper distribution of social benefits and burdens among society’s members*” [7]. (p. 16) This includes the fair distribution of resources, including wealth, income, rights, material resources, opportunities, power, and privilege, with the understanding that social, economic, and political institutions should equally benefit all groups in society [7]. Reimer Kirkham and Browne contextualize social justice by explaining that, “*while justice has to do with fairness, social draws our attention to the application of justice to social groups, and as in the case of population-based health, brings into focus how justice and injustices are sustained through social institutions and social relationships, highlighting the embeddedness of individual experience in a larger realm of political, economic, cultural and social complexities*” [8] (p. 325). Social justice perspectives, then, are particularly attuned to understanding differences between *groups*, pointing toward an underlying collectivist ideology rather than the individualist ideology pervasive in Western societies [7,8,9].

Understood as the absence of unfair and avoidable differences in health among and between population groups, health equity is closely linked to social justice. Sen [10] asserts that health equity is a “*central feature of the justice of social arrangements*” (p. 659). In other words, health equity cannot exist in the absence of social justice. Conversely, health inequities are both a product of, and a reflection of, unfair social, economic, and political arrangements [11,12]. Because of their nature as avoidable and unnecessary, health inequities are manifestations of social *in*justices, and ought to be both morally and ethically concerning. Nurse scholars have argued that social justice ideals have long been central to the profession [5,6], although how these ideals have been enacted has not always been clear. As oncology nurses practicing in diverse roles and contexts, we see social justice as both a lens through which to understand health and illness, and as a guiding principle that grounds our practice.

## 3. Understanding Cancer as an Issue of Social Justice

Using social justice as a lens through which to view health inequities, a social justice perspective draws attention to how cancer risk, access to care, and outcomes are shaped by structural inequities. Globally, groups who experience structural marginalization (i.e., are rendered marginalized and vulnerable as a result of the intersecting impacts of social, economic, political, and historical factors) are significantly more likely to be diagnosed with cancers that are preventable, to be diagnosed with late-stage disease (including for cancers with well-established screening programs), receive poor quality or no treatment for their cancer, experience poor pain and symptom management, and die from cancers that are generally curable [13,14,15,16,17,18,19,20]. For example, Indigenous women in Canada experience a cervical cancer incidence rate three times higher than the general population, and a mortality rate four times higher than the general population, despite a publicly funded healthcare system, for a cancer that is almost entirely preventable, and often curable if detected early [21]. In the USA, there is significant evidence of race-based inequities in cancer stage at diagnosis and cancer survival outcomes [22,23]. In North America, people experiencing homelessness and housing instability have an overall higher incidence of cancer, more advanced cancers at diagnosis, and cancer-related mortality rates twice as high as the general population [24]. If we consider equitable health outcomes as evidence of social justice, we can also understand these inequities as examples of social *in*justice.

## 4. Inequities Across the Cancer Continuum as Examples of Social Injustice

Evidence of health inequities in the form of inequitable cancer outcomes and access to care exists across the cancer continuum, ranging from screening and early detection to palliative and end-of-life care. Although from a social justice perspective, we are most interested in differences in health *outcomes*, there are also significant inequities in access to cancer care, and strong evidence that access to care plays a role in differences in cancer outcomes between groups, particularly in the case of access to early detection and cancer treatment [25]. In this section, we walk through the cancer continuum, highlighting social injustices in the form of inequities in cancer outcomes and access to cancer care. Our intention is not to provide an exhaustive or systematic review, but to provide a starting point for thinking about differences in cancer outcomes and access to care differently—as issues of equity and social justice. We primarily draw on evidence from North America to support our analysis; however, emerging evidence suggests that cancer-related inequities are a global phenomenon, but may manifest differently in various geographical regions [26].

***Screening.*** People impacted by the intersecting impacts of poverty, unstable housing, racism, discrimination, and stigma (related to substance use, mental health, and gender or sexuality) experience significant barriers to cancer screening at both the healthcare provider and health system levels, resulting in lower rates of screening for many cancers [27,28,29,30]. Histories of individual trauma, sexual violence, or historical and intergenerational trauma, and distrust in healthcare providers or institutions further compound barriers to cancer screening [31,32,33]. For example, women who experience homelessness are known to have very low rates of cervical cancer screening, despite having a higher incidence of cervical cancer [31]. This reflects the sobering reality that women experiencing homelessness are struggling to meet daily needs for food, shelter, and income, have poor access to health and social services, experience multiple forms of stigma, and often have past or ongoing experiences of trauma and violence, which all impact their ability to access cervical cancer screening and their willingness to engage in screening that holds the possibility for re-traumatization [31]. This represents a double-edged sword of sorts, in which experiences of health and social inequities put people at greater risk of cancer by limiting their access to the resources for health, while simultaneously limiting their access to healthcare services that promote health.

***Diagnosis.*** Groups experiencing socioeconomic disadvantage (e.g., poverty) and racialization (e.g., Black and Indigenous people) are known to be at greater risk of being diagnosed with advanced cancers [23,34,35]. For example, low-income women in the USA were found to be less likely to be diagnosed with breast cancer through mammography, and more likely to be diagnosed with late-stage breast cancer than higher-income women [35]. Conversely, in Canada, evidence suggests that those with *higher* income are more likely to be diagnosed with breast cancer at an *earlier stage* [36]. Beyond cancer stage at diagnosis, there is evidence of differences in experiences of cancer diagnosis. For example, Sinding et al. describe how patients with serious mental health challenges report having physical symptoms dismissed as symptoms of their mental illness and not investigated in a timely way, delaying their eventual diagnosis of cancer [37]. Similarly, racism and discrimination often result in patients being dismissed and not taken seriously, and weeks, months, or even years later diagnosed with metastatic disease [38,39]. In the case of cancer diagnosis, it appears that disadvantage perpetuates disadvantage, whereby people who experience disadvantage (by way of socioeconomic status, “race”, or stigma, for example) are further disadvantaged by way of barriers to accessing diagnostic care, leading to later-stage cancer diagnosis and, subsequently, poorer cancer outcomes.

***Treatment.*** Socioeconomic status, unstable housing, racism, and stigma variably impact access to cancer treatment, receipt of cancer treatment (including guideline-recommended treatment and timely treatment), and patient experiences of treatment. Although often assumed to be a function of health insurance and access to financial resources, there is growing evidence that low socioeconomic status has significant impacts on cancer treatment. This exists even in countries with publicly funded healthcare systems, including referrals to radiation and medical oncologists after cancer diagnosis, receipt of timely treatment, and receipt of guideline-recommended treatment [15,39,40,41,42]. Systemic racism seems to play a significant role here as well. For example, we know that patients who do not identify as White are more likely to encounter delays in treatment for prostate cancer, receive poorer-quality treatment, and experience more side effects after treatment for prostate cancer than patients who identify as White [23]. Evidence also indicates that racism, discrimination, and stigma result in patients whose pain and symptoms are inadequately assessed and/or managed during cancer treatment [17,37,43].

***Survivorship.*** Significant disparities in cancer survival are observed among groups who are racialized [22,23,44], socioeconomically disadvantaged [13,18,41], and otherwise marginalized within society [45]. Those who do survive cancer are faced with a host of disparities in long-term effects of cancer treatments and inequities in access to survivorship care. For example, damage to the cardiovascular system, a common sequala of cancer treatment, has been demonstrated to be worse in cancer survivors who have lower income, who are less educated, and who are Black [46]. Cancer survivors who are considered medically underserved are also at higher risk for worse health-related quality of life, a measure of physical, psychological, social, and financial well-being [46]. Compounding these outcomes is evidence of inequitable receipt of surveillance and psychosocial care among cancer survivors considered underserved, with barriers to accessing survivorship care exacerbated [47,48].

***Palliative and End-of-Life Care.*** Inequities in access to palliative and end-of-life care exist for groups who are racialized, socioeconomically disadvantaged, or who live in rural or remote geographies [22,49]. The WHO reports that globally, only 14% of people who need palliative care have access to it [50]. People who experience extreme poverty and housing instability are often unable to access palliative and end-of-life care until very late in their disease trajectory, if at all [51], and as a result, die alone and unsupported of cancers that are preventable and/or treatable [52,53].

## 5. Discussion and Implications

“*Social justice then provides a moral compass that refocuses us to see beyond an individualistic perspective—a vantage point that ultimately leaves us oblivious to the many dimensions of oppression, and that prompts us to continually intervene in a partial manner, rarely addressing root causes of inequities and disparities. Without the integration of critical perspectives into our social justice discourses, we are left in the positions where we facilitate adaptation to current unjust social structures rather than any effective address of issues such as poverty, systematic diminishment of life opportunities (participation as full citizens), and health disparities*”.

[8] (p. 337)

Our brief review of inequities in cancer-related health outcomes, access to care, and research has only scratched the surface of the growing body of evidence documenting pervasive and widening cancer-related inequities. These inequities are evidence of social injustices because they are a result of unfair social arrangements, and are amenable to intervention. As nurses—advocates, clinicians, researchers, health system administrators, and policymakers—we ought to be concerned about such injustices and prepared to take meaningful action. Yet, in our own work and spheres of influence, we have often noted limited action, or action that is merely tokenistic, paying lip service to addressing inequities but avoiding tackling the underlying causes of inequity. A recent scoping review underscored our experiences, with findings suggesting that little meaningful action is being taken within the cancer care sector to redress these inequities [54].

In considering the implications and directions for nursing (the noun), perhaps a starting place would be to return to our understanding of what it means to do nursing (the verb). As scholars, clinicians, and advocates, we draw on Reed’s conceptualization of nursing processes as the *facilitation of well-being, inherent within human systems* [55]. Although this has predominantly been co-opted to mean wellness in *physiological* systems (i.e., nursing’s role in pursuing homeostasis among the organ systems of a person’s body in an institutionalized hospital setting), we posit that nursing’s domain of concern equally extends to other systems that fundamentally shape the health and wellness of people, families, communities, and societies, including our health and social care systems. Even when nursing processes are directed at an individual level, we must never divorce ourselves from the context in which individuals live, grow, work, and play, and the health inequities that arise from within those contexts, including those arising from colonialism, racism, sexism, and other systems of oppression. More importantly, we are in a position to witness the human suffering that results from these systems of oppression and their impacts on the everyday lives and health of individuals and families, which means we have both a moral and ethical responsibility to take action [56,57,58].

Returning now to the concept of social justice, considering this broadened understanding of what it means to nurse, social justice offers a framework for not only understanding cancer inequities as rooted in systemic and structural inequities (rather than primarily in lifestyle and behavioural “choices”), but also direction for where and how to begin to redress these inequities. As nurses, social justice can also serve as a guiding principle that drives our practice, advocacy, and research. As Reimer Kirkham and Browne argue above [8], taking a social justice lens to our understanding of cancer-related inequities will focus our attention on the root causes of inequities. By taking what we (now) know about cancer as a social justice issue (including our experiential knowledge, clinical observations, extant research, and theory) and applying that knowledge to advance social justice through health equity, we are engaging in nursing *praxis*.

In considering what actions we might take, a starting place is to identify where your voice can be a vehicle for change within your specific practice context. As nurses, we can contribute to social justice and health equity through broadening our understanding of health to include the social and structural determinants of people’s health and healthcare access as it relates to our specific practice contexts, and advocating for this broader understanding of health within the systems we work in. Through collective advocacy and by recognizing our strength in numbers as the largest body of healthcare providers, we can call for equitable access to the resources necessary for health, and equitable access to healthcare. In our specific practice contexts, we can advocate for and work towards collecting the types of data that are necessary to understand the existing and ongoing inequities in health outcomes and access to resources, while also using that data to continually monitor our progress toward redressing these inequities. Nurse scholars can design research that exposes social injustices and seeks to co-develop strategies to advance health equity. Developing power literacy—an understanding of how power operates, where it is held, and how to challenge power—is critical to enacting social justice and redressing health inequities (for a comprehensive discussion, see McGibbon, 2024) [59]. In our practice contexts, we can address unequal relations of power: at the micro level, by demonstrating allyship in our interactions with patients, families, and other healthcare providers. At the meso level, we can partner with communities who already know what they need, and support their access to resources; we can continually ask ourselves and our organizations *who* our service programs are designed to serve, and whom they *are not.* At the macro level, we can ensure that nurses are included at policy and planning tables at every level and are equipped with the critical leadership competencies necessary to advance health equity [59].

## 6. Conclusions

Applying a social justice lens to our understanding of cancer and cancer inequities helps us to understand how power, privilege, and systems of oppression filter down to impact the lives, health, and healthcare experiences of the people whom we have the opportunity to care for as nurses. At the same time, a social justice lens illuminates a path forward—one that focuses on changing and transforming the systems and structures resulting in such inequities, rather than focusing exclusively on developing interim and band-aid solutions [60]. As a profession that has long held social justice as a core value, it is time that we fully enacted those values. We fully recognize that there are and will be significant challenges along the way, not the least of which will be questions of whether we can “afford” to tackle such wicked problems; the bigger question is: can we afford not to?
